# The development and validation of a prognostic model to PREDICT Relapse of depression in adult patients in primary care: protocol for the PREDICTR study

**DOI:** 10.1186/s41512-021-00101-x

**Published:** 2021-07-02

**Authors:** Andrew S. Moriarty, Lewis W. Paton, Kym I. E. Snell, Richard D. Riley, Joshua E. J. Buckman, Simon Gilbody, Carolyn A. Chew-Graham, Shehzad Ali, Stephen Pilling, Nick Meader, Bob Phillips, Peter A. Coventry, Jaime Delgadillo, David A. Richards, Chris Salisbury, Dean McMillan

**Affiliations:** 1 Department of Health Sciences, University of York, York, England; 2 Hull York Medical School, University of York, York, England; 3 Centre for Prognosis Research, School of Medicine, Keele University, Keele, England; 4 Centre for Outcomes and Research Effectiveness, Research Department of Clinical, Educational and Health Psychology, University College London, London, England; 5 iCope – Camden and Islington Psychological Therapies Services, Camden & Islington NHS Foundation Trust, London, England; 6 School of Medicine, Keele University, Keele, England; 7 Department of Epidemiology and Biostatistics, Schulich School of Medicine & Dentistry, Western University, London, ON Canada; 8 Camden & Islington NHS Foundation Trust, St Pancras Hospital, London, England; 9 Centre for Reviews and Dissemination, University of York, York, England; 10 Department of Psychology, University of Sheffield, Sheffield, England; 11 Institute of Health Research, College of Medicine and Health, University of Exeter, Exeter, England; 12 Department of Health and Caring Sciences, Western Norway University of Applied Sciences, Inndalsveien 28, 5063 Bergen, Norway, USA; 13 Centre for Academic Primary Care, University of Bristol, Bristol, England

**Keywords:** Relapse, Recurrence, Depression, Prognosis, Prognostic model, Predictive model

## Abstract

**Background:**

Most patients who present with depression are treated in primary care by general practitioners (GPs). Relapse of depression is common (at least 50% of patients treated for depression will relapse after a single episode) and leads to considerable morbidity and decreased quality of life for patients. The majority of patients will relapse within 6 months, and those with a history of relapse are more likely to relapse in the future than those with no such history. GPs see a largely undifferentiated case-mix of patients, and once patients with depression reach remission, there is limited guidance to help GPs stratify patients according to risk of relapse. We aim to develop a prognostic model to predict an individual’s risk of relapse within 6–8 months of entering remission. The long-term objective is to inform the clinical management of depression after the acute phase.

**Methods:**

We will develop a prognostic model using secondary analysis of individual participant data drawn from seven RCTs and one longitudinal cohort study in primary or community care settings. We will use logistic regression to predict the outcome of relapse of depression within 6–8 months. We plan to include the following established relapse predictors in the model: residual depressive symptoms, number of previous depressive episodes, co-morbid anxiety and severity of index episode. We will use a “full model” development approach, including all available predictors. Performance statistics (optimism-adjusted C-statistic, calibration-in-the-large, calibration slope) and calibration plots (with smoothed calibration curves) will be calculated. Generalisability of predictive performance will be assessed through internal-external cross-validation. Clinical utility will be explored through net benefit analysis.

**Discussion:**

We will derive a statistical model to predict relapse of depression in remitted depressed patients in primary care. Assuming the model has sufficient predictive performance, we outline the next steps including independent external validation and further assessment of clinical utility and impact.

**Study registration:**

ClinicalTrials.gov ID: NCT04666662

## Introduction

Depression is the leading cause of disability worldwide [1], and most people with depression are treated in primary care [2]. Around half of patients will experience a re-emergence of depressive symptoms at some point after their initial symptoms have improved, and for the majority of these, this occurs within the first 6 months [3]. Relapse and recurrence are both terms used to describe the re-emergence of depressive symptoms following some level of improvement. Generally, relapse occurs after some improvement (remission) but before full recovery [4], whereas recurrence is the onset of a further, separate episode after full recovery. While there is no empirically derived temporal cut-off to distinguish relapse from recurrence, recovery is most commonly operationalized as following an extended period of remission; between 6 and 12 months [5]. Relapse, then, occurs within 6–12 months, while recurrence occurs beyond 6–12 months [4, 6].

The distinction between relapse and recurrence provides a useful theoretical framework and there may be some clinical relevance. The implication is that the re-emergence of symptoms in relapse is part of the unsuccessfully treated index episode of depression, while in recurrence, it is attributable to a new and separate episode of depression. When the MacArthur Foundation Research Network defined these terms, or “change points,” in 1991 [4, 6], their aim was to provide a framework that might be more consistently applied in the empirical literature, but also that the framework and definitions themselves be validated empirically by researchers. There have been limited attempts to do this, though where this has been attempted researchers have found some evidence to support their validity [7]. Given the wide variability in the way in which the terms relapse and recurrence have been operationalized by researchers, however, Bockting et al. [5] suggested using the terms interchangeably to describe the “re-emergence of symptoms following a period of relative wellness”. We will use the term *relapse* throughout this paper.

There is some evidence that the severity of depression [8] and risk of further relapse [9–11] increases with each subsequent depressive episode, highlighting the potential benefits of intervening early to prevent relapse and recurrence with a view to improving the overall trajectory of depression. Efforts to prevent relapse could be improved by an increased ability to predict prognosis and identify high-risk individuals. Prognosis can be shaped by multiple factors, and once it is established which factors are associated with an outcome, the information can be used to create a multivariable prognostic model. Prognostic models aim to provide individualised risk estimates for a specified outcome by a particular time conditional on the individual’s values for multiple prognostic factors (or predictors) [12]. We currently lack evidence-based tools to assist clinicians with risk predictions of depressive relapse.

There have been some previous attempts to develop relapse prediction models for depression [13–17]. These pre-existing prognostic models have some drawbacks with respect to successfully predicting relapse in a primary care context. Critical appraisal of these studies, using the Prediction model Risk Of Bias Assessment Tool (PROBAST), found that the majority of these studies were at high overall risk of bias [18]. The most significant limitations were inadequate sample size, inappropriate handling of missing data and presentation of inappropriate performance statistics (calibration and discrimination not assessed) [18]. Furthermore, the developed models have either demonstrated insufficient predictive performance on external validation [13], or they could not be feasibly implemented in a primary care setting due to the large number and type of included predictors [16].

This protocol outlines the methods for the development and validation of a novel prognostic model to predict an individual’s risk of relapse of depression in a primary care setting. The long-term aim, beyond this study, is to implement the prognostic model in clinical practice for use by primary care health professionals to enable optimal shared decision making with patients. The model must, therefore, be accurate, generalisable and effective (i.e. result in demonstrably improved outcomes for patients). In order to be implemented in practice, it must also be clinically credible and have face validity to healthcare professionals and patients.

### Objective

The objective is to develop and validate a multivariable prognostic model to predict relapse within 6 to 8 months in patients with remitted depression in primary care.

## Methods

The methods have been developed in accordance with those recommended by the PROGnosis RESearch Strategy (PROGRESS) initiative [19, 20], and the prognostic model will be published according to the Transparent Reporting of a multivariable prediction model for Individual Prognosis or Diagnosis (TRIPOD) Statement [21]. This study will use individual participant data (IPD) from RCTs and a cohort study; therefore, elements of the Preferred Reporting Items for a Systematic Review and Meta-analysis of Individual Participant Data (PRISMA-IPD) statement are also relevant [22]. However, this study is not a systematic review and the aim is not to provide a summary of a complete body of research and so not all items are applicable. A Patient and Public Involvement (PPI) group of service users have informed several aspects of this study, including selecting predictors and their measurement (for example, commenting on the acceptability of validated diagnostic instruments for depression and anxiety symptoms), definition of outcome, target patient population and clinical application. The study has been registered prospectively on ClinicalTrials.gov (available: ClinicalTrials.gov ID: NCT04666662).

### Source of data

We have formed a cohort using IPD from UK primary care-based datasets. Along with cohort studies, RCTs are a recommended source of data for development of prognostic models [23]. We had IPD readily available in a pragmatic sample of four RCTs (CASPER Plus [24], REEACT [25], REEACT 2 [26] and COINCIDE [27]), derived from RCTs carried out within our own research group. In order to increase the sample size available for model development, we identified further studies: first, by searching all the National Institute for Health Research (NIHR)-funded RCTs of primary care-based interventions for depression, and second, by reference to the search results from a recent IPD meta-analysis of RCTs of depression interventions (this meta-analysis had searched for studies that had used the CIS-R as a measure of baseline severity and provided a recent search of relevant studies) [28].

To be included, we specified that RCTs must:

- Include adult patients (18 years and over) with depression and measure depressive symptoms at a minimum of three time-points (to enable diagnosis of depression, remission, relapse/no relapse). We excluded RCTs in patient groups with significant psychiatric or medical comorbidity. We also excluded feasibility studies (due to limited sample size and shorter follow-up time associated with those identified);
- Have sufficient follow-up to allow us to detect relapse within at least 6 months;
- Use only non-pharmacological interventions (e.g. psychological, social, behavioural). We excluded RCTs of pharmacological interventions, as these were felt likely not to be comparable to the pharmacological interventions that patients would be receiving from their primary care healthcare providers as usual treatment. Trials of pharmacological interventions often use medication combinations that would not be routinely prescribed in primary care and would therefore potentially reduce the generalisability of the model. Non-pharmacological interventions are more likely to affect outcomes in a comparable way;
- Use the Patient Health Questionnaire (PHQ-9) as a measure of depression.

This search added three further RCTs: COBRA [29], CADET [30] and Healthlines Depression RCT [31].

Finally, we contacted the authors of the West Yorkshire Low Intensity Outcome Watch (WYLOW) study, a longitudinal cohort study following-up patients after low-intensity cognitive behavioural therapy (LiCBT) through the Improving Access to Psychological Therapies (IAPT) service [3]. See Table 1 for details of the final included studies.

**Table 1:** Summary of primary sources of IPD

Study	N	Study type	Inclusion criteria	Length of follow-up	Follow-up points	Mean age (SD)	Gender (% female)	RCT intervention	Duration of RCT intervention
CASPER Plus	485 (358 at 12 m)	RCT	65 years or older with depression	18 months	0, 4, 12 and 18 months	Intervention group: 71.9 (6.03) Control: 71.6 (5.96)	Intervention group: 59.1 Control: 63.1	Collaborative care	8–10 weeks
REEACT	461	RCT	Adults with depression	24 months	0, 4, 12 and 24 months	39.86 (12.65)	67	cCBT	6 weeks
REEACT 2	369	RCT	Adults with depression	12 months.	0, 4 and 12 months	40.6 (13.8)	64.5	cCBT	4 months
COINCIDE	387	RCT	Adults with depression and multi-morbidity	24 months	0, 4, 12, 24	58.5 (11.7)	38	Collaborative care	3 months
COBRA	440	RCT	Adults with depression	18 months	0, 6, 12, 18	43.5 (14.1)	66	Behavioural activation vs CBT	16 weeks
CADET	581	RCT	Adults with depression	12 months	0, 4,12	44.4 (13.3)	71.9	Collaborative care	14 weeks
Healthlines Depression	609	RCT	Adults with depression	12 months	0, 4,8,12	Intervention group: 49.1 (12.9) Control: 50 (12.8)	Intervention group: 69 Control: 68	Complex intervention (integrated telehealth)	12 months
WYLOW	439	Longitudinal observational cohort study	Adults with depression	12 months (Start-point = Remission)	Monthly	41.28 (14.59)	59.7	None (cohort are followed-up after LiCBT)	NA

All of the included studies had pragmatic and unrestrictive inclusion criteria, and so are expected to be representative of the target population. The final PREDICTR dataset is derived from all arms (control and intervention) of seven randomised controlled trials (RCTs) of low-intensity primary care-based interventions for depression (CASPER Plus, REEACT, REEACT 2, COINCIDE, CADET, COBRA, Healthlines) and one observational cohort study (WYLOW).

### Participants

Adult participants (aged 18 years and over) with depression. The included participants do not have significant psychiatric comorbidity (e.g. schizophrenia, bipolar affective disorder).

### Setting

All data sources are primary care or community-based.

### Start-point (remission)

There are three important time-points: baseline for the RCT (i.e. the point at which patients were depressed); follow-up 1 (FU1; to diagnose remission; t=0 for our prediction model study and corresponds with a 4-month follow-up for RCTs) and follow-up 2 (FU2; the intended prediction time and occurs at either 6 or 8 months after t=0; patient either relapses or does not relapse).

In all RCTs, the majority of participants are expected to meet criteria for a diagnosis of depression at baseline. Any participants identified to have a baseline PHQ-9 less than 10 will be excluded from the analysis. As described, FU1 is required to detect “remission” and FU2 to detect “relapse/no relapse.”

The start-point (or time of intended prediction) is FU1, the point at which a patient, who started treatment with case-level depression, has entered remission. The PHQ-9 is a screening tool for major depressive disorder and a cut-off of 10 or more is used to detect clinically significant depressive symptoms [32]. Remission will be identified as a participant who had case-level depression at baseline (a PHQ-9 score of 10 or more) having (i) a post-treatment PHQ-9 score below the established cut-off of 10 at 4 months after trial baseline (this is consistent with clinical recovery [30] as currently operationalized in the NHS Improving Access to Psychological Therapies (IAPT) service [3]) and (ii) an improvement of ≥5 points on the PHQ-9 (which aligns with the established reliable change index used to identify those with “reliable improvement” [33]).

### End-point (relapse)

Patients will be coded as relapsed if they fulfil the following criteria within 6 to 8 months post-remission: (i) PHQ-9 score above the diagnostic cut-off (10 or more) and (ii) ≥5 points greater than their symptom score at the time of remission. As above, this is consistent with accepted criteria for reliable and clinically significant deterioration [33, 34].

The main reason for specifying the prediction end-point at 6 to 8 months rather than a single-time point is pragmatic and based on the available data (the time between FU1 and FU2 is 8 months for six of the seven RCTs and 6 months for COBRA). As discussed in the “Introduction” section, relapse is most commonly operationalized as occurring between 6 and 12 months post-remission [35] and the majority of patients who do relapse do so within the first 6 months [3]. Relapse by 6–8 months is felt to be an appropriate and sufficiently short-term timeframe for predictions to be meaningful and clinically useful for patients and primary care professionals.

### Predictors

We identified predictors based on literature review and on clinical grounds through discussion of a multidisciplinary group including members of the research team and the PPI group supporting the project. Umbrella reviews (reviews of other systematic reviews and meta-analyses) are one of the highest levels of evidence for determining associations between predictors and outcomes when selecting predictors for inclusion in a prognostic model [36]. A recently published umbrella review of prognostic factors associated with increased risk of relapse and recurrence guided the selection of candidate predictors for inclusion in the model [37]. A further systematic review of prognostic factors, published after the umbrella review, supported those findings and was also used to guide our included predictors [38]. In addition to this, we reviewed all existing prognostic models for predicting relapse or recurrence to explore other predictors used [18]. All candidate predictors are based on self-report or clinical information, and we have not included, for example, biomarkers and in-depth neuropsychological testing in an effort to ensure that the model is acceptable and usable in a primary care setting [39].

All included studies have information about key predictors, measured using reliable and validated tools. See the “Missing Data” section for details of how missing predictor information will be handled. Categorisation of continuous predictors will be avoided in order to avoid loss of information and power to detect an association between predictors and outcomes [19].

The following variables have robust evidence for their role as relapse predictors and will be included in the model (Table 2).

**Table 2:** Summary of selected predictors

Predictor	Type of data	Method of measurement	Range of values and coding of predictors
PHQ-9 score at point of remission (residual depressive symptoms)	Continuous	PHQ-9 score at remission (t=0)	Range from 0 to 9
Number of previous episodes of depression	Categorical	Patient or GP report (No previous episodes vs any previous episodes)	No previous episodes=0; 1 or more previous episodes=1
Comorbid anxiety	Continuous	GAD-7 Score	0–21
Severity of episode	Continuous	PHQ-9 score at baseline	10–27
RCT intervention	Categorical	Presence or absence of effective treatment	Remission after receiving control=0; Remission after receiving intervention=1

### PHQ-9 score at remission (residual depressive symptoms)

Residual depressive symptoms is a strongly established predictor of relapse [37, 38] and will be operationalized in this study using the Patient Health Questionnaire (PHQ-9) score. The PHQ-9 is a validated tool for screening and case-finding for depression [32], routinely used in primary care. Remission is defined as a PHQ-9 score below 10 (remission), and residual symptoms are defined as a PHQ-9 at remission of between 5 and 9 [33]. Per the inclusion criteria for this study, all participants will meeting criteria for remission (i.e. PHQ-9 score of below 10); PHQ-9 score at remission (0–9) will be modelled as a continuous variable rather than binary (e.g. presence or absence of residual symptoms).

### Number of previous episodes of depression

There is strong evidence that this is a significant predictor [37, 38], albeit slightly less strong than for residual symptoms. We plan to model this as a dichotomous predictor. The coding of this variable in the original RCTs is variable (i.e. a combination of continuous and dichotomous), and so it would not be possible to model as a continuous variable in this study. While there is some weak evidence that the relapse risk increases with each successive depressive episode, the prognostic effect of previous episodes on recurrence is strongest when comparing any number of previous episodes to no previous episodes [37]. This finding from the pre-existing literature is likely to be helpful for a primary care-based prognostic model, as there is potential difficulty in achieving a precise number of previous episodes in clinical practice. In this study, we will model this predictor as a dichotomous variable (0=no previous episodes, 1=one or more previous episodes) and will accept patient report, GP report or documentation in GP records.

### Comorbid anxiety

There is good evidence that comorbid anxiety predicts relapse or recurrence of depression and will be included as a predictor in the model [37, 38]. The GAD-7 is a valid tool for screening and assessing severity of Generalised Anxiety Disorder score in clinical practice [40]. Pre-treatment symptoms (i.e. those at baseline) seem to be more predictive of relapse than those at depressive remission [38]. The pre-treatment GAD-7 score will be used provided it is available for all datasets; otherwise, we will use the GAD-7 at remission (t=0). GAD-7 score will be modelled as a continuous predictor.

### Severity of episode

There is reasonable evidence that the baseline severity of the *index* episode is a prognostic indicator of greater odds of relapse [37]. This will be measured using the PHQ-9 score at baseline (pre-treatment) rather than that at the point of prognostication (remission). The PHQ-9 score at the point of depression diagnosis will be modelled as a continuous predictor.

### RCT intervention

Because the data are drawn from RCTs, we must be mindful of the fact that approximately half of the participants have received a treatment (above usual care) and the other half have not. Where the treatments were found to be effective, not modelling the effect of different treatments can lead to unreliable risk predictions when the model is validated in a different population. Excluding the treated individuals would mean losing half of the available data, and so a preferable option is to explicitly model for treatment effect when developing a prognostic model [41, 42]. The treatments in all RCTs were acute-phase psychological treatments rather than relapse prevention interventions, and therefore, we do not know what their effect on relapse outcomes were. One of the studies [31] did include an element of relapse prevention beyond the acute phase treatment (advisors phoned the patients every 2 months to check how they were getting on and encourage them to keep following the intervention advice). The interventions are also heterogeneous, and so it is possible that they affected relapse outcomes in different ways. To avoid overcomplicating the model, we will code the presence or absence of an effective intervention as a dichotomous variable. We will define an effective intervention by whether individual participants entered remission after receiving an RCT intervention (code=1) or whether they entered remission after receiving a control (code=0).

### Exploratory predictors

We also plan to conduct an exploratory analysis investigating the role of the following less well established predictors: age; gender; ethnicity; employment status; relationship status; and multi-morbidity (Table 3). Age, gender and ethnicity are not well supported by the pre-existing evidence as being associated with relapse [37, 43, 44], but are routinely collected during RCTs and often included as predictors in prognostic models [19]. There is weak evidence that employment status [45] and relationship status [46, 47] may be associated with an increased risk of relapse or recurrence. The National Institute for Health and Care Excellence (NICE) defines multi-morbidity as the presence of two or more long-term mental or physical health conditions [48]. The extant literature suggests that this is not associated with an increased risk of relapse or recurrence [44, 49]. The exploratory predictors described here are relevant to a primary care setting and, therefore, will be investigated outside of the planned principal analysis, depending on the completeness of the data and final sample size.

**Table 3:** Summary and categorisation of exploratory predictors

Variable	Type of data	Method of measurement	Original categories (in RCTs)	New categories (PREDICTR)	Range of values and coding of predictors
Age	Continuous	RCT data collection/self report	Not applicable	Not applicable	
Gender	Categorical	RCT data collection/self report	Unchanged	Unchanged	Men=0; Women=1
Ethnicity	Categorical	RCT data collection/self report	White	White	White=0; Other=1
			Mixed	Other	
			Black		
			Asian		
			Chinese		
			Other		
Employment status	Categorical	RCT data collection/self report	Employed (Full time or part time)	Employed/not seeking employment	Unemployed=0; Employed or not seeking employment=1
			Student		
			Retired		
			House-person		
			Other		
			Unemployed job-seeker	Unemployed	
			Unemployed due to ill-health		
Relationship status	Categorical	RCT data collection/self report	Married/civil partnership/cohabiting/relationship	In a relationship	Single=0; In a relationship=1
			Single	Single	
			Separated		
			Divorced		
			Widowed		
Multi-morbidity	Categorical	RCT data collection/self report	None	No long-term physical health condition	No long-term physical health conditions=0; One or more long-term physical health conditions=1
			Mental health only		
			Diabetes	One or more long-term physical health conditions	
			Asthma or COPD		
			Degenerative or inflammatory arthritis		
			Heart Disease		
			Stroke		
			Cancer		
			Kidney Disease		

### Sample size

Ensuring an adequate sample size will allow for more accurate estimation of regression coefficients and reduce the potential for overfitting. Rules of thumb for calculating required sample size for prediction models with binary outcomes (such as ten events per candidate predictor parameter (EPP)) are now considered too simplistic to provide robust estimates of minimum required sample size [50]. The actual required sample size is context-dependent and is informed by several factors. We used the *pmsampsize* package in Stata (available online: https://riskcalc.org/pmsamplesize/) to calculate our required minimum sample size [51].

The Cox-Snell R^2^ is a measure of overall model fit and based on the method of Riley et al. [51] an anticipated Cox-Snell R^2^ must be specified when calculating sample size, usually based on previous studies of similar patient groups/outcomes. No previous prognostic model study predicting relapse of depression identified so far has reported a Cox-Snell R^2^ and so, to ensure an adequate minimum sample size, we used the recommended conservative estimated Nagelkerke R^2^ of 15% [52]. This corresponds to a Cox-Snell R^2^ of 0.0945, assuming an overall outcome proportion of 0.2, which again is a conservative estimate based on the literature [3]. We targeted an expected shrinkage factor (*S*) of 0.9 (to reflect small optimism in predictor effect estimates), as recommended [52].

To include all predictors, we require 8 predictor parameters (*P*), which corresponds to PHQ-9 score at remission, previous depressive episodes, co-morbid anxiety, severity of index episode and RCT Intervention (including 2 parameters for each continuous predictor to account for potential non-linear trends). Therefore, our minimum required sample size (*n*) is 722 (with 145 events) for these predictors. Our actual sample size exceeds this, and therefore, we anticipate that the study will be of a sufficient size to require minimal shrinkage and provide meaningful estimates of predictive performance.

### Missing data

To avoid loss of power and precision, missing data will be handled using multiple imputation with chained equations (MICE) [53]. Missing values will be imputed based on other predictor and outcome values, under a missing at random assumption, and multiple copies of the dataset will be created with identical known information and different imputed values, reflecting the uncertainty associated with imputation. Imputation will be undertaken for each RCT separately, to preserve the clustering of participants within trials and any between-trial heterogeneity in predictor effects and outcome prevalence. We will assume that data are missing at random, unless this appears inappropriate upon inspection and discussion with original trialists. We will use the percentage of participants with one or more missing values to determine the number of imputations needed, in line with current guidance; at least 20, as long as this is greater than or equal to the percentage of participants with one or more missing values [19, 54]. Results from non-imputed and imputed data will be compared as a form of sensitivity analysis. Given the selection criteria, we do not anticipate any systematically missing predictors across datasets.

### Statistical analysis methods

#### Data pre-processing

The datasets will be combined and harmonised to ensure consistency across trials. To assess IPD integrity, we will compare numbers of participants in each treatment arm with those reported in the primary references. We will check the relapse rate within each arm and compare these across datasets. To define the quality of the IPD for prognostic modelling, we will perform risk of bias assessment on the included datasets using the PROBAST [55]. Only the participants, predictors and outcome domains are pertinent; the analysis domain is used for assessment of prognostic model development and validation studies which do not apply to the RCTs included in this study.

Once remission has been identified this will represent time t=0. Relapse will then be coded as 0=no relapse, 1=relapse as described in “End-point” section. Descriptive statistics will be produced for all predictors and outcome data. Exploratory univariable analysis will be performed to evaluate the unadjusted relationship between each predictor variable and the outcome variable, but not for the purpose of informing predictor selection. We will explore percentage of cases that relapse over the different studies to assess comparability of data sources.

#### Model development

The model will be developed using a multilevel multivariable logistic regression, with a binary (relapse/no relapse) outcome. Model parameters will be estimated via unpenalised maximum likelihood estimation and then penalised post-estimation using a uniform shrinkage factor (see later). The modelling will preserve the clustering of patients within trials, by having a random effect on the intercept, a random intervention effect and a random control effect, also allowing for between-study correlation in these pair of effects. If it is not possible to fit random effects in the multilevel logistic regression model, as originally planned, we will explore alternative modelling approaches. This would initially consist of a Generalised Estimating Equation model to control for the clustering without a random intercept. If this is also not possible, we will perform a single-level analysis with robust standard errors and accept that the limitation is that there may be a clustering effect that we are unable to properly control for.

Stepwise methods for predictor selection are not generally recommended for prediction models as this has been reported to remove judgment of the analyst from the process of model development as well as leading to *estimation bias* (estimating the performance of a prediction model after testing for statistical significance of predictors in the same data) [36]. We have selected our key predictors on the grounds of best available evidence and clinical acceptability, as well as practical reasons related to data availability. The list of predictors is felt to be of appropriate length, so we will avoid predictor selection techniques during model development and include all predictors regardless of their statistical significance (“full model” approach) [56]. This will also apply in the presence of multi-collinearity, which is not an issue for prediction purposes. We will only consider the need to exclude predictors due to collinearity if this is preventing convergence of the estimated model.

The full model approach described has the advantage of not being overly data-dependent and avoids the risk of removing clinically important predictors from the final model [56]. Calibration plots with loess smoothed calibration curves will be provided. Optimism will be measured and adjusted for using bootstrapping.

We will explore non-linear relationships in the modelling process using multivariable fractional polynomials (MFPs), a flexible and recommended approach for modelling continuous predictors in medical datasets. The other recommended method for modelling continuous predictors is the use of restricted cubic splines, and while these two methods often result in similar models, there is some evidence that MFPs perform better than restricted cubic splines in the presence of simpler relationships and medium amounts of information, as is the case here [20, 57]. We have factored in two predictor parameters (beta coefficients) per continuous variable to account for this approach, as described in the “Sample size” section.

Beyond the primary analysis outlined, and dependent on final sample size, an exploratory analysis will be performed investigating the role of less established relapse predictors (Table 3). Univariable associations between these predictors and outcome will be explored and, because the role of these variables as relapse predictors is less well understood, predictor selection through stepwise backward elimination will be used to develop an exploratory model. With sufficient sample size, stepwise backward elimination is an acceptable form of variable selection, performs similarly to other predictor selection approaches (for example, LASSO [58]) and is more compatible with our planned approaches for handling missing data and exploring non-linear trends. Guidance suggests using a more liberal p-value than the standard 0.05 for retention [19]; we will use a p-value of 0.157 or less as a stopping rule (consistent with Akaike information criteria (AIC) at one degree of freedom) [59].

#### Internal validation

The predictive performance and optimism of the developed model will be assessed. Calibration (a measure of the agreement between predictions from the model and observed outcomes) will be assessed by plotting observed vs predicted risks for groups defined by tenths of individual predicted risk (calibration plot) and by including a loess smoothed calibration curve across individuals (avoiding grouping). Apparent and optimism-adjusted calibration-in-the-large and calibration slope will be estimated. Discrimination (the ability of the model to differentiate between those who do or do not relapse) will be assessed using the C (concordance) index. The C-index assesses the extent to which the model assigns a higher probability of relapse to a patient who did eventually relapse in contrast to a patient who did not. The optimism-adjusted C-index will be derived using bootstrapping.

Optimism describes the risk of obtaining misleading measures of predictive accuracy when this is assessed in the same dataset used for model development, mainly due to overfitting. Internal validation can be used to provide optimism-corrected performance statistics can mitigate for this effect. Non-parametric bootstrapping will be used as a means of resampling the original dataset. This has the advantage, for example over a single split-sample approach, of allowing all of the data to be used in model development. Bootstrapping will be performed within each individual study, and then, these will be combined to create a new bootstrap sample to ensure studies are represented evenly for the final analysis. Multiple imputations for missing data will be performed within each bootstrap sample.

A bootstrap sample will be created in which the model development process will be repeated. The performance of this model will be evaluated in the bootstrap sample (bootstrap, or apparent, performance) and in the original sample (test performance). This process will be repeated hundreds of times and the average difference between the bootstrap and test performance for each performance statistic provides the estimate of optimism for that statistic. Optimism-adjusted performance statistics will be derived by subtracting the average optimism estimate (from bootstrapping) from the apparent performance *of the original model*. The uniform shrinkage factor (calculated as the optimism-adjusted calibration slope) will then be applied to all estimated predictor effects to produce a penalised logistic regression model, and the intercept updated to ensure calibration-in-the-large.

Sensitivity, specificity and positive and negative predictive values for the model will be calculated at risk thresholds considered potentially clinically relevant. It is unclear whether the creation of risk groups is in the best interests of patients, but they are often used to guide clinical decision making [21]. In the absence of a gold standard test (as is the case here), the need for and definition of risk groups will be determined based on discussion within the research team and through consultation with our PPI group during model development. We will avoid basing risk thresholds on the data used to develop the model. The net benefit of the model at particular thresholds will also be examined using decision curve analysis and compared to treat all and treat none decisions [60].

#### External validation

External validation is the assessment of a model’s predictive performance in data not used in the development process and is a measure of a model’s generalisability and performance in a range of populations and settings. To conserve information and to allow for all data to be used for model development, we do not plan to perform a conventional external validation as part of this study. We do, however, have IPD from multiple studies, and therefore, generalisability and heterogeneity of the model performance will be examined using internal-external cross-validation (IECV) [61], as follows. We will exclude data from each primary study in turn and develop the risk prediction model using the remaining data. We will then externally validate the developed model using the data from the excluded study. This process will be repeated, each time omitting a different study, until the model has been fitted excluding each study once. Random effects meta-analysis will then be used to summarise the performance across studies, to obtain summary measures of the model performance and estimates of heterogeneity in performance across studies. We will ensure that each cycle of the IECV approach retains sufficient sample size for model development; in this manner, each cycle will retain the majority of the available IPD for model development, and so the final models produced in each cycle are likely to be similar to each other. A consistent model development strategy will be used in each cycle of the IECV approach [62].

A sensitivity analysis will be performed measuring predictive performance statistics omitting, first, the observational cohort data (WYLOW) and, secondly, the RCT (COBRA) with relapse at 6, rather than 8, months. If our risk of bias (PROBAST) assessment identifies any studies that are not at overall low risk of bias, we will also perform a sensitivity analysis omitting these studies.

## Discussion

We have reported a protocol for the development and validation of a novel prognostic model to predict depressive relapse in a primary care setting. As discussed, we have used an up-to-date review of the extant literature to guide predictor selection and our sample size is in excess, relative to the number of predictor parameters, of those used in previous prognostic model studies. We now briefly discuss our anticipated next steps, beyond this prognostic model study.

It is envisaged that this statistical model could form the basis for a clinical tool, to be embedded in GP IT systems, to help identify patients who are at higher risk of relapse. Longer term, and with further research, a decision tool could be developed to help inform decisions as to which patients with remitted depression should receive relapse prevention interventions. Provided we are able to demonstrate sufficient predictive accuracy during the validation stages, the model should undergo external validation (in a different dataset, to assess generalizability) and, ideally, independent validation (by a different research team, to reduce risk of bias). External validation could be done on either an unrelated retrospective dataset or, preferably, a prospective dataset collected specifically for this purpose. Finally, the impact of the model should be evaluated, and the gold standard way of doing this is through a randomised controlled trial with clinically meaningful outcome measures [63].

Qualitative work with stakeholders will be used to decide the extent to which the model can be implemented and will guide the evaluation of the model in practice, including plans for impact assessment. In particular, Cuijpers recently highlighted the importance of assessing the effect of mental health treatments on patient-defined outcomes (e.g. quality of life and functional outcomes) as well as those determined to be important by researchers or clinicians [64]. This is applicable to health technologies, like prognostic models, and exploring patient-defined outcomes will form a part of our evaluation process beyond this study.

### Limitations

The ideal dataset for developing a prognostic model is a prospective, pre-designed cohort study. The advantage of such an approach is that investigators retain control over inclusion and exclusion criteria, definition and measurement of predictors and outcomes, ensure appropriate timings, reduce missing data and minimize other potential biases (for example, selecting bias and blinding). However, the costs (financial and time) of carrying out a prospective study would be substantial and secondary analysis of good quality data from RCT and other cohorts is an accepted alternative [23]. We are mindful of the potential problems with this approach, particularly the risk of missing data (that we have planned for) and the chance that predictors and outcomes may not be recorded optimally. We are reliant on the quality of the initial data collection with respect to this latter point, and we are confident that the studies included are of a high standard.

A further common pitfall of RCTs is the narrow eligibility criteria often stipulated which can impact on the generalizability of any findings to the target population of interest (in our case, a primary care patient population). We are reassured that the eligibility criteria for included studies were inclusive and pragmatic with relatively small numbers of participants with missing data. We do however recognise that RCT participants may differ from the general population in important ways and results should be interpreted with this in mind.

In the planning stage of this project, we considered other data sources, in particular the Clinical Practice Research Datalink (CPRD), a large electronic database of routinely collected follow-up data from primary care. Following discussions with CPRD experts at the University of York, it was evident that the coding of measures of relapse and recurrence were not optimal for identifying patients who relapsed and that this would have limited our ability to develop a reliable and generalisable model.

Further limitations relate to measurement of start-point (remission) and end-point (relapse or not), which will be measured using PHQ-9 score. The gold standard would have been to use diagnostic interviews, which may have been possible with a prospective cohort study. The PHQ-9 is a validated and widely used tool with good sensitivity and specificity [65], and the large sample size (possible because of the use of secondary data analysis) should compensate for this. A further point to consider is that the start- and end-points are defined at the next time-point they were actually measured rather than necessarily capturing the precise “real-world” moment of remission. However, this reflects the situation in general practice, where diagnostic tools will be applied at patient consultation rather than in real time. Therefore, we feel this is justifiable and actually mirrors the clinical picture accurately. We will use the reliable clinical recovery and deterioration definitions (sample size allowing) to ensure robust coding of start- and end-points.

In the event that multilevel modelling with a random intercept and random effects on the intervention/control variable is not possible, we will be required to make an assumption that the effects of the different interventions and controls in the RCTs were homogenous. It is not likely that the interventions had a significant effect on relapse rates, even where they did improve acute depression symptoms. However, it is possible that one or more of the interventions (or controls) did exert an effect on relapse of which we are not aware. We will take a pragmatic approach to modelling this, following the steps that we have outlined in the “Methods” section. A further limitation is that the data we plan to analyse do not allow for survival analysis, as the follow-up time-points were insufficiently similar and infrequent. However, time to relapse is important and would increase our understanding; future prospective work should consider this when designing strategies for data collection.

There are some predictors not included due to lack of relevance and usefulness to GPs. For example, neuroticism (the personality trait), childhood maltreatment and rumination have been found to be associated with increased risk of relapse and recurrence [37], as has duration of index episode of depression and age at onset of first episode of depression [66]. These are not routinely measured in practice and have not been coded for in our cohorts; they will therefore not be included as predictors. The cohort has been designed to be as undifferentiated as possible to represent a GP case-mix. Increased predictive performance would be more likely if we were to be very specific in defining this cohort, but this would have implications for its utility in the real-world primary care setting.

In summary, this study will derive a statistical model aiming to predict relapse. If it demonstrates sufficient predictive performance, it could be used to guide the allocation of interventions to prevent relapse in a primary care setting, improving outcomes for patients and ensuring efficient use of healthcare resources.

## Data Availability

Not applicable.

## Abbreviations

CADET: The Clinical and Cost-Effectiveness of Collaborative Care for Depression in UK Primary Care Trial
CASPER: CollAborative care for Screen-Positive EldeRs
CBT: Cognitive behavioural therapy
cCBT: Computerised cognitive behavioural therapy
COBRA: Cost and Outcome of Behavioural Activation versus Cognitive Behaviour Therapy for Depression
COINCIDE: Collaborative Interventions for Circulation and Depression
GAD: Generalised anxiety disorder
GP: General practitioner
IPD: Individual participant data
LiCBT: Low-intensity cognitive behavioural therapy
MFP: Multivariable fractional polynomials
PHQ-9: Patient Health Questionnaire
PROBAST: Prediction Model Risk Of Bias Assessment Tool
RCT: Randomised controlled trial
REEACT: Randomised Evaluation of the Effectiveness and Acceptability of Computerised Therapy
WYLOW: West Yorkshire Low-intensity Outcomes Watch
